# Distinct cytokine pattern in aqueous humor during immune reactions following penetrating keratoplasty

**Published:** 2010-01-15

**Authors:** Philip Maier, Ulrike Heizmann, Daniel Böhringer, Yvonne Kern, Thomas Reinhard

**Affiliations:** University Eye Hospital and Lions Cornea Bank Baden-Württemberg, Freiburg, Germany

## Abstract

**Purpose:**

Cytokine patterns in the aqueous humor before penetrating keratoplasty might permit us to predict the development of immune reactions. We therefore analyzed ten different cytokines in the aqueous humor of patients before and following penetrating keratoplasty.

**Methods:**

We collected aqueous humor from patients undergoing cataract extraction or penetrating keratoplasty (baseline, n=197), from patients undergoing cataract extraction after penetrating keratoplasty without signs of an endothelial immune reaction (acceptors, n=33), and from patients who underwent irrigation of the anterior chamber due to endothelial immune reactions (rejectors, n=25). We determined interleukin (IL)-1b, IL-2, IL-4, IL-5, IL-6, IL-8, IL-10, IL-12, interferon (INF)-γ, and tumor necrosis factor-α in the aqueous humor, using cytometric bead arrays.

**Results:**

We found statistically significant differences for IL-10 and INF-γ among the study groups. We observed no significant differences concerning all other cytokines evaluated in this study.

**Conclusions:**

Elevated levels of INF-γ emphasize its important role in immune reactions following penetrating keratoplasty. Antagonizing INF-γ might be an option when treating immune reactions. High levels of IL-10 during endothelial immune reactions may reflect a downregulation of the delayed-type hypersensitivity. Perhaps this is a donor-specific activation of the immune privilege in the anterior chamber as an attempt to prevent damage during immune reactions. We plan to investigate, in upcoming prospective trials, whether the analysis of cytokine patterns in the aqueous humor before penetrating keratoplasty will enable us to predict the occurrence of immune reactions.

## Introduction

Good long-term prognosis of penetrating corneal grafts has been attributed to the immunological privilege of the cornea and anterior chamber [1]. In addition to transforming growth factor (TGF)-β_2_, supposedly the most important immunosuppressive cytokine in this context, various other immunosuppressive as well as immunostimulatory cytokines play a role in maintaining this privilege.

Corneal graft rejection, however, remains the most important reason for graft failure [2]. Despite intensive research, the exact mechanisms leading to graft rejection have not been fully understood. Considering the immunosuppressive climate in the anterior chamber, the contents of specific cytokines might correlate with the development of immune reactions or long-term, clear, graft survival. One assumption is that T helper cells 1 (Th1) type cytokines, such as interleukin-2 (IL-2), interferon γ (INF-γ), or tumor necrosis factor α (TNF-α), are more likely to be associated with allograft rejection than Th2-type cytokines, such as IL-4, IL−5, IL−6 or IL−10 [3].

We have observed that active [4] but not total [5] TGF-β_2_ is reduced during endothelial immune reactions. Moreover, we recently found that active TGF-β_2_ is increased in the aqueous humor of keratoconus patients before penetrating keratoplasty (PK), which might be one reason for the excellent graft prognosis in those patients [6].

As a result of a newly developed technology, namely microparticle-based flow cytometric analysis [7], we were able to determine six different cytokines simultaneously from only 50–100 µl of aqueous humor. In this study we aimed to discover whether the expression patterns of ten cytokines (IL-2, IL-4, IL-5, IL-6, IL-8, IL-10, IL-12, TNF-α, and INF-γ) would differ between patients before cataract surgery or PK (baseline) and patients with (rejectors) or without (acceptors) endothelial immune reaction following PK.

## Methods

### Patients

All patients included in this study were recruited following reference to the University Eye Hospital Freiburg by their treating ophthalmologists. Group 1 (baseline, n=197) comprised patients who underwent cataract extraction (n=94) or PK for various indications (n=103, with 30 cases of Fuchs endothelial dystrophy, 12 of herpes simplex virus keratitis, 20 of bullous keratopathy, 28 of keratoconus, 5 of corneal scars, and 8 with various other indications). Mean patient age in this group was 66 years, 53% of patients were male and 47% were female.

Group 2 patients underwent cataract extraction following PK with no signs of an endothelial immune reaction (acceptors, n=33). Mean patient age in this group was 58.5 years, 36% of patients were male and 64% were female.

Group 3 (rejectors) included 25 patients following PK presenting with a newly diagnosed endothelial immune reaction. After aqueous humor sampling, the anterior chamber was irrigated with corticosteroids for therapeutic reasons in those patients. As a therapeutic irrigation of the anterior chamber was only necessary in severe and manifest cases of endothelial immune reactions, we could include only this stage of endothelial immune reactions. Mean patient age in this group was 48.1 years, 48% of patients were male and 52% were female.

All invasive procedures were performed following written informed consent in adherence to the Declaration of Helsinki for research involving human subjects. Research was approved by our ethics committee of the Albert-Ludwigs-University, Freiburg, Germany. Detailed information on patients is provided in Table 1.

**Table 1:** Graft and patient data of all study groups.

**Clinical parameter**	**Baseline**	**Acceptors**	**Rejectors**	**p Value**	**Statistical test**
Mean donor age/years (n)	61.3 (95)	66.4 (17)	57 (14)	0.31	ANOVA
Mean interval from death to graft excision/hours (n)	28.5 (95)	30.1 (17)	22.2 (14)	0.4	ANOVA
Duration of organ culture/days (n)	21.5 (93)	21.8 (17)	19.3 (14)	0.51	ANOVA
Preoperative endothelial cell density of the graft/cells per mm^2^ (n)	2,684 (93)	2,782 (16)	2,435 (14)	0.32	ANOVA
Patient age/years (n)	66 (197)	58.5 (33)	48.1 (25)	<0.01	ANOVA
Patient gender female/% (n)	47 (197)	64 (33)	52 (25)	0.20	χ^2^

n=number of patients with data available/applicable.

All keratoplasty procedures were performed under general anesthesia and all cataract surgeries and anterior chamber irrigations were performed under topical anesthesia.All keratoplasties were performed with mechanical trephines (either hand guided trephines made by Geuder, Heidelberg, Germany or by a guided trephine system GTS®, Polytech Ophthalmologie GmbH, Rossdorf, Germany). To fix the grafts we used a double running cross-stitch suture with nylon 10.0 [8]. Gentamycine ointment (Refobacin®, Merck Serono GmbH, Darmstadt, Germany) was administered following surgery at least until the graft had been covered by a complete epithelial layer. Prednisolone-21-acetate 1% eye drops (Prednipos®, Ursapharm GmbH, 66129 Saarbrücken, Germany) were then given five times daily and tapered during the first 5 postoperative months. Systemic corticosteroids (Urbason®, Sanofi-Aventis GmbH, Frankfurt, Germany) were administered for only 3 weeks postoperatively. Oral acetazolamide (Diamox®, Goldshield pharmaceuticals, Croydon, UK) was administered at a daily dose of 2×125 mg for 5 days postoperatively.

All grafts were preserved in organ culture according to the guidelines of the European Eye Bank Association. Detailed graft data are provided in Table 1. The endothelial immune reactions of the group 3 patients were diagnosed with a slitlamp via endothelial precipitates and stromal edema.

Anterior chamber puncture was performed within 24 h after the patients’ referral to the clinic. All eyes were rinsed with sterile solution (BSS^®^, Alcon Pharma GmbH, Freiburg, Germany) before anterior chamber puncture. A paracentesis lancet was used to penetrate the cornea in an avascular peripheral area over a length of 1 mm. Contact with limbal or peripheral corneal vessels was completely avoided or the samples were discarded. Aqueous humor (0.05–0.1 ml) was drawn into conventional tuberculine syringes without coming into contact with intraocular structures.

The cytokine levels in each sample were measured using a cytometric bead array (CBA kit; BD Bioscience, San Diego, CA). Briefly, 50 µl of samples was added to a mixture of 50 µl each of capture antibody-bead reagent and detector antibody-phycoerythrin (PE) reagent. The mixture was subsequently incubated for 3 h at room temperature in the dark, and washed to remove unbound detector antibody-PE reagent. The experimental procedure is described elsewhere [9]. Data were acquired by flow cytometry (FACSCalibur™ Flow cytometer, BD Biosceinces, Franklin Lakes, NJ). For our analyses we used the human inflammation kit (n=163 for baseline, n=12 for acceptors, and n=10 for rejectors) for determining IL-1b, IL6, IL-8, IL-10, IL-12, and TNF-α and the Th-1/Th-2 kit (n=34 for baseline, n=21 for acceptors, and n=15 for rejectors) for determining IL-2, IL-4, IL-5, IL-10, TNF-α, and INF-γ. All values were calculated with regards to the negative control in each assay. If the fluorescent signal of a sample was equal to or below the fluorescence of the negative control, the cytokine level was set as 0 pg/ml.

For statistical analysis all computations were performed using the R-software system [10]. Comparison of the cytokine levels among the three study groups was made using the Kruskal–Wallis test for each of the cytokines. Holm’s multiple comparison adjustment provided corrected p values for all cytokines. The level of confidence at which the adjusted results were judged significant was p<0.05. The R functions used were “kruskal.test” and “mt.rawp2adjp” of package multtest.

To test whether the cytokine profiles would enable us to classify patients as baseline, acceptors, or rejectors, we used a recently developed tree classification algorithm, random forest [11]. This method grows a collection of decision trees constructed from different bootstrap samples of the data. Each node is split, using the best among a subset of predictors randomly chosen at that node. The random forest algorithm estimates the importance of a variable by examining how much prediction error increases when data for that variable are permuted while all others are left unchanged. The necessary calculations are performed tree by tree as the random forest is constructed. The R-function used was “randomForest” in the random Forest package.

## Results

**Figure 1:**
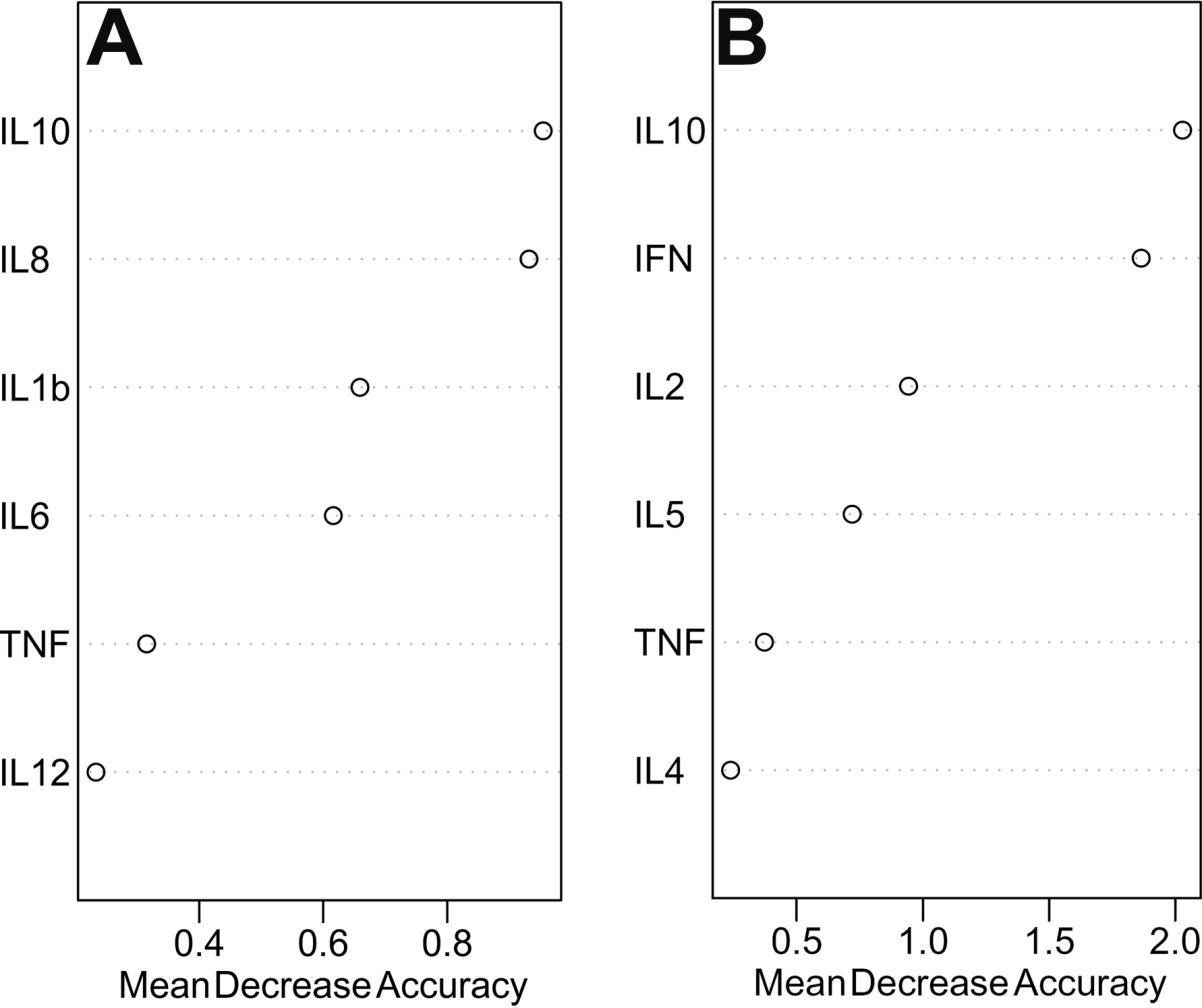
Random forest analysis demonstrates the relative importance of individual cytokines in classifying different study groups.Random forest analysis of data set 1 (**A**) and data set 2 (**B**) demonstrating the relative importance of individual cytokines in classifying aqueous humor from different study groups. The individual plots represent the relative importance of the cytokines in the overall classification. The vertical axes represent individual cytokines sorted by importance. The horizontal axes represent the average decrease in classification accuracy. Abbreviations: IL=interleukin, TNF=tumor necrosis factor, INF=interferon.

The random forest algorithm estimates the importance of a variable in discriminating among study groups. Therefore, we first performed this analysis on both data sets to identify which cytokines seemed key to permitting differentiation among study groups. The results of these analyses are demonstrated in Figure 1, which illustrates the relative importance of individual cytokines in classifying aqueous humor from different study groups.

Random forest analysis of both data sets showed that IL-10 seems to be the most important cytokine that might allow classification of the study groups. The second most important cytokines were IL-8 for data set 1 and INF-γ for data set 2.

**Figure 2:**
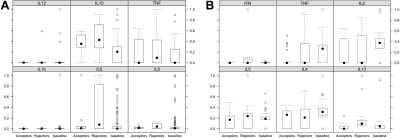
Box plot diagram shows the relative cytokine levels.Box plot of the relative cytokine levels on data set 1 (**A**; human inflammation kit) and data set 2 (**B**; Th-1/Th-2 kit). Relative cytokine levels were determined by dividing the individual cytokine level through the maximum cytokine level of that cytokine measured in the whole study group. After correction for multiple comparisons, the Kruskal–Wallis test revealed a statistically significant difference only for IL-10 in data set 1 and for INF-γ in data set 2 among the three study groups. The filled dots represent median values, the circles outliers, and the boxes 95% intervals. Abbreviations: IL=interleukin, TNF=tumor necrosis factor, INF=interferon.

Using the Kruskal–Wallis test for each of the cytokines and Holm’s multiple comparison adjustment, we observed no statistically significant difference among the three study groups in the levels of IL-1b, IL-2, IL-4, IL-5, IL-6, IL 8, IL-12, and TNF-α. However, IL-10 was statistically significantly different among the three groups (group 3 mean 5.58 pg/ml, median 3.65 pg/ml; group I mean 2.05 pg/ml, median 1.85 pg/ml; group 2 mean 2.02 pg/ml, median 1.6 pg/ml; p<0.001). Furthermore, we found statistically significant (p=0.03) INF-γ levels between group 3 (mean 22.58 pg/ml, median 0 pg/ml), group 1 (mean 1.22 pg/ml, median 0 pg/ml), and group 2 (mean 0 pg/ml, median 0 pg/ml; Figure 2).

The results of individual cytokine analysis for differences among study groups concur, in part, with the random forest analysis results as we found that IL-10, INF-γ, and IL 8 were the three most important cytokines for differentiating among study groups. Due to a lack of statistical power following corrections for multiple testing, we could not find statistically significant differences for IL-8 between study groups, although IL-8 seemed to be important for differentiating the study groups in the random forest analysis. The detailed results for all cytokine levels are summarized in Table 2.

**Table 2:** Detailed information on cytokine levels.

**Study group**		**Cytokine (pg/ml)**									
		**Il-1b**	**IL-2**	**IL-4**	**IL-5**	**IL-6**	**IL-8**	IL-10*	**IL-12**	**TNF-α**	INF-γ*
Baseline (group 1, n=197)	Mean±SD	24.94±230.7	1.42±0.99	1.85±1.15	1.13±0.86	484.9±1136	64.00±180	2.17±3.31	11.27±75.56	0.88±1.32	1.49±2.85
	median	0	1.5	1.8	1.1	18.0	10.3	1.85	1.3	0	0
	(range)	(0–2,943.5)	0–4.0	0–5.7	0–3.9	(0–5,000)	(0–1,148)	(0–29.1)	(0–806.7)	(0–6.7)	0–11.6
	n=0	89	8	5	8	17	12	70	78	125	21
	n	163	34	34	34	163	163	197	163	198	34
Acceptors (group 2, n=33)	Mean±SD	4.53±5.65	0.69±1.02	1.17±0.99	0.81±0.87	541.0±1,423	24.24±18.84	1.92±2.2	2.64±2.3	0.78±1.36	0
	median	1.95	0	1.5	1.0	49.75	18.95	1.6	2.6	0	0
	(range)	(0–15)	(0–2.6)	(0–2.5)	(0–2.9)	(3.5–5,000)	(5.0–72.9)	(0–7.8)	(0–5.6)	(0–4.8)	na
	n=0	6	14	8	10	0	0	16	4	23	21
	n	12	21	21	21	12	12	33	12	33	21
Rejectors (group 3, n=25)	Mean±SD	7.86±16.21	1.07±1.33	1.15±1.28	1.37±1.51	1,772.4±2.171	61.87±53.78	5.80±7.11	49.54±151	1.21±1.88	24.09±68.23
	median	1.45	0	1.2	1.4	385.8	47.55	3.65	2.2	0	0
	(range)	(0–52.7)	0–3.4	0–4.0	0–5.90	(0–5,000)	(4.5–151.2)	(0–32)	(0–479.3)	(0–7.5)	0–267.9
	n=0	5	8	7	5	1	0	2	4	15	9
	n	10	15	15	15	10	10	25	10	25	15

The asterisk indicates a statistically significant difference compared to group 3. na=not applicable. n=0 means the number of samples with a cytokine level below the detection limit, which were set as zero in the statistical analysis.

We found a statistically significant difference among the three groups regarding age, with the youngest patients belonging to group 3 (rejectors). However, we observed no statistically significant correlation between age and any of the cytokine levels.

## Discussion

In this study we aimed to identify whether cytokine expression patterns differ between patients before PK and patients following PK with or without endothelial immune reaction. We not only observed statistically significant differences of IL-10 and INF-γ levels in the aqueous humor of patients with an endothelial immune reaction compared to baseline patients and graft acceptors but also found that IL-10 and INF-γ levels played the most essential role in differentiating among study groups following random forest analysis. We observed no statistically significant differences in the levels of IL-1b, IL-2, IL-4, IL-5, IL-6, IL-8, IL-12, and TNF-α among the study groups, which led us to conclude that the roles of those cytokines during an endothelial immune reaction are less important. We plan to examine whether all these cytokines have a prognostic impact on the development of immune reactions in long-term prospective studies of the same patient cohort.

In the statistical analysis we observed that the age distribution in the three study groups differed significantly, with the youngest patients belonging to group 3. This is not surprising as the older patients in groups 1 and 2 had undergone cataract extraction, whereas group 3 included only patients with an endothelial immune reaction following PK. As we found no statistically significant correlation between age and any of the cytokines we evaluated (data not shown), the data we report are most likely not confounded by age inhomogeneity. In contrast to TGF-β_2_, whose levels drop in older patients [12], age does not seem to significantly influence the levels of the cytokines determined in this study.

Surprisingly, we did not find higher but rather somewhat lower levels of the pro-inflammatory, Th1-related, cytokine IL-2 in the aqueous humor of patients with endothelial immune reaction compared to controls. As IL-2 is believed to be a barrier to tolerance, thus leading to immune reactions [13], higher IL-2 levels during an immune reaction were anticipated, and therefore our findings contradict the Th-1/Th-2 paradigm. However, IL-2 is not necessary for allograft rejection as IL-2 knockout mice revealed only modestly reduced survival rates [14]. Moreover, corneal endothelial cells were shown to inhibit T-cell proliferation by blocking IL-2 production in vitro [15]. We also found that antagonizing IL-2 by basiliximab in a pilot study was less effective in preventing endothelial immune reactions than cyclosporin A [16]. Thus, the potential immune reaction-inducing effect of IL-2 on T-cell proliferation may be strong in vitro but somewhat less profound in vivo following PK. Another factor explaining low IL-2 levels may be the time point of aqueous humor analysis in this study as increased IL-2 levels might be responsible for the induction and the beginning of immune reactions but not for their maintenance when they are clinically visible, as was the case in study group 3.

We found no elevated IL-6 levels in the aqueous humor during an endothelial immune reaction. This is in contrast to the findings by Flynn et al. [17] as well as Funding et al. [18] who demonstrated that increased IL-6 levels during an endothelial immune reaction are a result of IL-6 production by the intraocular environment as a reaction to the rejection process and not a result of plasma influx. The reason for this incongruency is most likely the lack of statistical power from multiple comparisons in our study.

The increased levels of IL-10 during an endothelial immune reaction seem to stand in contrast to the functions of IL-10 in that it has primarily immunosuppressive effects by inhibiting the production of pro-inflammatory cytokines, such as IL-1, IL-2, or TNF-α. Furthermore, it has been shown that the gene transfer of IL-10 can lead to prolonged graft survival in different transplant models, including corneal transplantation [19,20]. However, Gong et al. [21] demonstrated that systemic but not local application of IL-10 gene vectors prolonged corneal graft survival. They concluded that IL-10 modulates cytokine expression in the draining lymph nodes, leading to the graft-protecting effect. The high IL-10 levels during an endothelial immune reaction found in this study might be a result of increased IL-10 production by the local environment in the anterior chamber. Therefore, a graft-protecting effect of these high IL-10 levels via the draining lymph nodes, as seen in systemic IL-10 treatment, could not be effective. As we further found that monocytes and macrophages are predominantly present in the aqueous humor during immune reactions [22,23], these cell types might be a possible source of IL-10 production during severe episodes of graft rejection. In this context another effect of IL-10, which is the upregulation of CD-163 expression on monocytes and macrophages [24], might also be important as the soluble form of CD-163 can be regarded as a marker for monocyte/macrophage activity [25]. As these cell types are found in the aqueous humor during severe immune reactions [23], locally increased IL-10 levels might also be responsible for increased levels of soluble CD-163 levels found in human aqueous humor during endothelial immune reactions [18]. It has been shown that increased levels of soluble CD-163 are the result of local CD-163 production and not of a systemic immunological response [18]. This further emphasizes the suspected locally increased production of IL-10 during graft rejection. Besides its anti-inflammatory effects, IL-10 is able to promote cytotoxic cell activity in vitro [26], and prolonged administration of IL-10 may have a detrimental effect on graft survival [27]. On the other hand, locally increased IL-10 levels may be interpreted as an attempt by the anterior ocular segment to restrict the inflammatory process by maintaining “some” immunological privilege in the presence of an acute inflammation. To solve the problem of these paradox IL-10 effects, further investigations regarding the local and systemic effects of IL-10 in the context of tissue and organ transplantation are necessary.

As INF-γ is a strong pro-inflammatory cytokine strengthening the Th-1 immune response, increased levels during the acute phase of an endothelial immune reaction do not seem surprising. This reinforces our findings that active TGF-β_2_, the counterpart of INF-γ, is statistically significantly decreased during an immune reaction [28]. However, the source of increased INF-γ levels in the aqueous humor during endothelial immune reactions remains unclear as Nicholls et al. [29] found no INF-γ-producing cells on corneal endothelium during immune reactions in animal experiments. This is further supported by the fact that we could not find INF-γ in the aqueous humor of nine out of 15 patients with active signs of an immune reaction. As this cannot be explained by clinical signs of the immune reaction, there must be some heterogeneity among the patients developing endothelial immune reactions following PK regarding the amount and probably the source of INF-γ production. Antagonizing INF-γ [30] or TGF-β_2_ gene transfer might be helpful in treating endothelial immune reactions in the future.

Random forest analysis revealed that, besides IL-10 and INF-γ, IL-8 seemed to be an important factor for the differentiation between the study groups. However, we could not find statistically significant differences in IL-8 levels between the study groups by using the Kruskal–Wallis test. The reason for this probably is the lack of statistical power from multiple comparisons in our study. It is not clear whether IL-8 plays any role in transplant immunology. However, as random forest analysis in our study revealed IL-8 to be important for differentiating between study groups, further research is necessary to identify its functions regarding rejection and acceptance in corneal transplantation.

King et al. [31] reported an early cytokine and chemokine response to the transplantation process in animal experiments (evident in syngeneic and allogeneic grafts) that probably drives angiogenesis, leukocyte recruitment, and affects leukocyte functions. Once an immune response is generated, allogeneic rejection results in the expression of Th-1 cytokines (IL-2, IL-12p40, IFN-γ), Th2 cytokines (IL-4, IL-6, IL-10, and IL-13), and anti-inflammatory Th3 cytokines (TGF-β1/2 and IL-1RA). When Funding et al. [32] determined 17 different cytokines (IL-1b, IL-2, IL-4, IL-5, IL-6, IL-7, IL-10, IL-12p70, IL-13, IL-17, TNF-α, and INF-γ), growth factors (granulocyte-monocyte colony-stimulating factor and granulocyte colony-stimulating factor), and chemokines (CXCL-8, monocyte chemoattractant protein-1, and macrophage inflammatory protein-1β) in the aqueous humor of patients with endothelial immune reaction, they found that all these factors were statistically significantly increased compared to patients with cataract or Fuchs’ endothelial dystrophy. This contrasts, in part, with the results we present here where we found no statistically significant differences for IL-1b, IL-2, IL-4, IL-5, IL-8, IL-12, and TNF-α. It is possible that our study’s larger number of baseline patients (n=163 for test 1, n=34 for test 2) led to the different results, as the control group in the study by Funding et al. [32] consisted of only nine patients and almost all the determined cytokine levels, except for IL-6 and IL-7, were below the detection limit in the aqueous humor of control patients. In contrast, with the exception of  INF-γ in group 2, we found each of the determined cytokines in the aqueous humor of patients in all study groups. In concurrence with the results of Flynn et al. [17] as well as Funding et al. [32], we observed no statistically significant differences between baseline and rejectors for IL-6 because we noted higher IL-6 levels in our control patients. Although neither Flynn et al. [17] nor Funding et al. [32] reported on a correction for multiple comparisons of their statistical analyses, their results would probably not be altered by doing so due to the very low p values. It seems unlikely that the use of different multiplex array kits by us and Flynn et al. [17] and Funding et al. [32] is responsible for the different results as the underlying testing technique is the same. However, in contrast to the results of both ours and Funding et al. [32], Flynn et al. [17] could not detect IL-2, IL-4, TNF-α, and INF-γ in the aqueous humor of patients with and without immune reaction following PK. This incongruence might be due to the larger sample size in our study. Another explanation could be that the definition of acute endothelial immune reaction, which is not defined in the study by Flynn et al. [17], was different so that less inflammation might have been present in their cases [17].

Finally, the source of the cytokines found in the aqueous humor of the patients presented in this study remains unclear. The source might either be cells already present or coming into the anterior chamber during an immune reaction or it might be a diffusion of systemic cytokines by a breakdown of the blood aqueous barrier. In an earlier investigation we showed that the isolation of immune cells (mainly macrophages and monocytes followed by lymphocytes) in aqueous humor is possible especially from patients with severe endothelial immune reactions [23], as were included in this study. However, additional cellular isolation from the small amount of aqueous humor gained from each patient was not possible. Furthermore, in former experiments the number of isolated cells was too small to perform further characterization, especially with respect to phenotyping or specific cytokine expression. Therefore, we aimed to determine whether a specific cytokine pattern in the aqueous humor would allow a conclusion on the immune cells (Th1 or Th2 cell) responsible for this respective cytokine expression, as discussed above for each cytokine. A systemic response to corneal transplantation in the peripheral blood regarding the number of immune cells or the cytokine expression is suspected to be very low as these effects were incongruent following cardiac transplantation [33]. Therefore, we waived this additional investigation in this study. However, following the results presented here, a more specific analysis (e.g., IL-10) in peripheral blood might help us understand the rejection processes following PK in the future.

Furthermore, as therapeutic irrigation of the anterior chamber was only necessary in severe and manifest cases of endothelial immune reactions, we only included this stage of the endothelial immune reactions. Therefore, from the results of this study we cannot comment or conclude on the influence of cytokine levels on the kinetics of endothelial immune reactions.

There are various other factors involved in the maintenance and regulation of the immunological privilege of the anterior chamber beyond the ten cytokines determined in this study. These factors might also play important roles in the occurrence and maintenance of endothelial immune reactions. Thrombospondin, for example, is a potent physiologic regulator of TGF-β activation [34], and somatostatin [35] as well as α-melanocyte-stimulating hormone [36] have been identified as important regulators of the anterior chamber-associated immune deviation. Thus, these factors must be taken into consideration in further aqueous humor analyses.

Our findings in this study might be interpreted as suggesting a primarily altered immunological privilege in eyes that develop immune reactions following PK. Future studies with long-term follow-up of this study’s baseline patients will likely reveal what happens to the cytokine levels over the long-term following PK and whether certain patterns correlate with fewer immune reactions and better long-term, clear, graft survival.
